# The Contribution of Singlet Oxygen to Insulin Resistance

**DOI:** 10.1155/2017/8765972

**Published:** 2017-09-07

**Authors:** Arnold N. Onyango

**Affiliations:** Department of Food Science and Technology, Jomo Kenyatta University of Agriculture and Technology, P.O. Box 62000, Nairobi 00200, Kenya

## Abstract

Insulin resistance contributes to the development of diabetes and cardiovascular dysfunctions. Recent studies showed that elevated singlet oxygen-mediated lipid peroxidation precedes and predicts diet-induced insulin resistance (IR), and neutrophils were suggested to be responsible for such singlet oxygen production. This review highlights literature suggesting that insulin-responsive cells such as endothelial cells, hepatocytes, adipocytes, and myocytes also produce singlet oxygen, which contributes to insulin resistance, for example, by generating bioactive aldehydes, inducing endoplasmic reticulum (ER) stress, and modifying mitochondrial DNA. In these cells, nutrient overload leads to the activation of Toll-like receptor 4 and other receptors, leading to the production of both peroxynitrite and hydrogen peroxide, which react to produce singlet oxygen. Cytochrome P450 2E1 and cytochrome c also contribute to singlet oxygen formation in the ER and mitochondria, respectively. Endothelial cell-derived singlet oxygen is suggested to mediate the formation of oxidized low-density lipoprotein which perpetuates IR, partly through neutrophil recruitment to adipose tissue. New singlet oxygen-involving pathways for the formation of IR-inducing bioactive aldehydes such as 4-hydroperoxy-(or hydroxy or oxo)-2-nonenal, malondialdehyde, and cholesterol secosterol A are proposed. Strategies against IR should target the singlet oxygen-producing pathways, singlet oxygen quenching, and singlet oxygen-induced cellular responses.

## 1. Introduction

Insulin resistance is a condition in which a given concentration of insulin produces a less than expected effect on target cells, and this may lead to impaired glucose tolerance ahead of overt type II diabetes mellitus [1]. It was recently reported that elevated plasma levels of products formed by singlet oxygen-mediated lipid oxidation precede and predicts the development of insulin resistance and diabetes in both humans and mice [2, 3]. Neutrophils recruited to adipose tissue as a result of high-fat feeding were speculated to be responsible for generating the singlet oxygen-modified lipids [2, 3]. On the contrary, the present article highlights literature consistent with (i) a primary role of insulin-responsive cells such as endothelial cells, adipocytes, hepatocytes, and skeletal muscle cells in singlet oxygen formation even prior to the activation of neutrophils and (ii) an important role of singlet oxygen in decreased insulin signaling by the insulin-responsive cells. Key insulin resistance-associated singlet oxygen-producing pathways in these cells are proposed, as well as mechanisms by which this ROS induces insulin resistance. New pathways are proposed for the singlet oxygen-mediated formation of bioactive aldehydes, including cholesterol secosterol aldehyde A, which was previously considered to be exclusively generated by cholesterol ozonolysis and to be a key piece of evidence for endogenous ozone formation.

## 2. Insulin Signaling

As reviewed by Siddle [4], insulin signaling begins with insulin binding to its receptor, a receptor tyrosine kinase, and this results in the sequential activation of (i) an insulin receptor substrate (IRS), typically IRS-1 or IRS-2, (ii) phosphatidyl inositol 3 kinase (PI3K), (iii) protein kinase B (PKB/Akt), and (iv) various Akt substrates such as the Akt substrate of 160 kDa, whose phosphorylation facilitates translocation of glucose transporter 4 (GLUT 4) from cytoplasmic storage vesicles to the plasma membrane of adipocytes and skeletal muscle cells. Akt-mediated phosphorylation of glycogen synthase kinase 3 results in the activation of glycogen synthase and enhanced glycogen synthesis, while Akt-mediated phosphorylation of the forkhead transcription factor (FOXO 1) prevents translocation of the latter to the nucleus and inhibits expression of enzymes responsible for hepatocyte gluconeogenesis and glycogenolysis [4]. In endothelial cells, Akt phosphorylates and activates endothelial nitric oxide synthase, leading to nitric oxide synthesis [5].

## 3. Obesity, Adipose-Derived Inflammation, and Insulin Resistance

Chronic adipose tissue inflammation during obesity promotes both adipose tissue and systemic insulin resistance, mainly through the release of proinflammatory compounds by various adipose tissue cells including adipocytes and macrophages, as well as neutrophils that were shown to infiltrate adipose tissue at an early stage of diet-induced obesity in mice [6, 7]. Nevertheless, there is evidence that such adipose tissue-derived inflammation is not essential for the initiation of systemic insulin resistance [8]. In a mouse model of diet-induced obesity, cellular inflammation and insulin resistance occurred first in arterial tissue, within the first week, followed by skeletal muscle and liver between weeks 4 and 8, and these changes were not detected in adipose tissue until week 14 [9]. Likewise, there was absence of systemic inflammation in grade 1 obese women, who displayed IR in skeletal muscle but not in adipose tissue [10]. At the cellular level, cultured endothelial cells, hepatocytes, or skeletal muscle cells treated with palmitate developed insulin resistance in the absence of neutrophils or macrophages [5, 11–14]. On the other hand, palmitate does not directly activate neutrophils, and it even reduces hydrogen peroxide production by these cells [15].

## 4. Toll-Like Receptor 4 or 2 (TLR4 or TLR2) Signaling in Response to High Fat, High Sugar, and Lipopolysaccharide Promotes Insulin Resistance through Oxidative Stress and the Activation of Serine Kinases

Oxidative stress refers to an imbalance between cellular reactive oxygen species (ROS) and antioxidants, in favor of the former [16]. Examples of ROS include superoxide anion (∙−O_2_), hydrogen peroxide (H_2_O_2_), hydroxyl radical (∙OH), singlet oxygen (^1^O_2_), and ozone (O_3_). There is mounting evidence that oxidative stress has a causative role in insulin resistance. For example, attenuating mitochondrial hydrogen peroxide emission by treating rats with a mitochondrial-targeted antioxidant or by overexpressing catalase in mouse skeletal muscle mitochondria preserved insulin sensitivity [17]. Excessive caloric intake by healthy men for 1 week acutely induced weight gain, oxidative stress, and insulin resistance in adipocytes prior to the onset of inflammatory stress [18].

**Figure 1:**
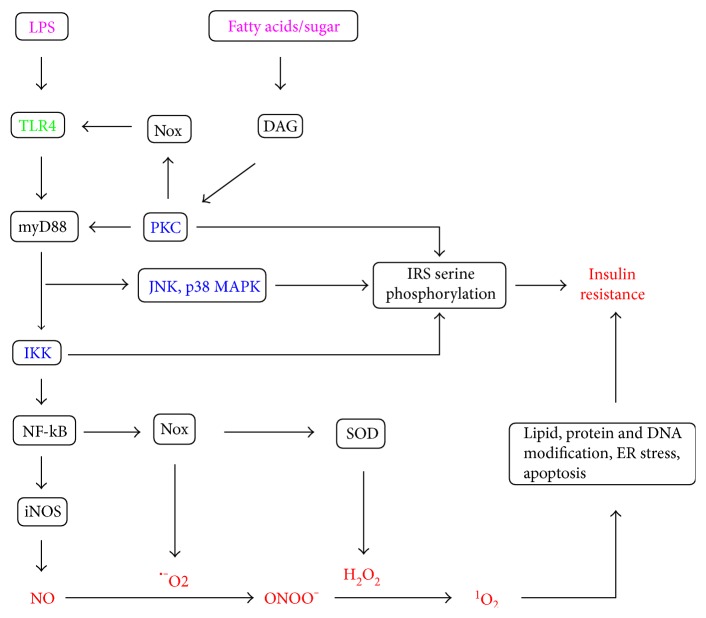
TLR4-dependent pathways for the development of insulin resistance via (i) the activation of serine kinases such as PKC isoforms, IKK, JNK, and p38 MAPK that induce serine phosphorylation of insulin receptor substrate (IRS) and (ii) the formation of reactive oxygen and nitrogen species such as hydrogen peroxide (H_2_O_2_), nitric oxide (NO), peroxynitrite (ONOO−), and singlet oxygen (^1^O_2_). Enteric lipopolysaccharide (LPS) is a direct ligand of TLR4, while fatty acids and sugars such as fructose activate this pathway via DAG, PKC, and Nox-derived ROS.

Toll-like receptor 4 (TLR4) signaling is responsible for much of the proinflammatory cytokine production by innate cells [19]. It is also expressed in other cells including endothelial cells, skeletal muscle cells, hepatocytes, and adipocytes, where it contributes to insulin resistance by promoting the formation of ROS and reactive nitrogen species such as nitric oxide (NO), as well as the activation of serine kinases which catalyze serine rather than tyrosine phosphorylation of IRS (Figure 1) [19, 20]. Such serine kinases include protein kinase C (PKC) isoforms, I*κ*B kinase (IKK) complex, and mitogen-activated protein kinases (MAPKs) such as c-Jun N-terminal kinase (JNK) and p38 MAPK [19]. p38 MAPK also activates phosphatase and tensin homolog (PTEN) which reduces phosphorylation of P13K and Akt [11].

Enterobacterial lipopolysaccharide (LPS), whose plasma levels are increased by a high-fat diet, is a direct ligand and activator of TLR4 through its lipid A component which partly consists of palmitic, lauric, or myristic acids [19, 20]. On the other hand, fatty acids and sugars may interact indirectly with TLR via diacylglycerol (DAG) synthesis and subsequent PKC and NADPH oxidase (Nox) activation (Figure 1), as discussed shortly. TLR signaling requires adaptors such as myeloid differentiation primary response protein 88 (myD88) and TIR-domain-containing adapter-inducing interferon-*β* (TRIF) [19, 21]. Both TLR2 and TLR4 signal via the myD88 pathway whereby myD88 leads to activation of TAK 1, a member of the MAPK kinase family, which activates both the IKK complex-nuclear factor kappa B (NF-kB) pathway and MAPKs such as ERK1/2, JNK, and p38 [21]. IKK activation leads to phosphorylation of the NF-kB inhibitor, IkB*α*, whose subsequent proteosomal degradation frees NF-kB to translocate to the nucleus and activate the expression of proinflammatory cytokines as well as inducible nitric oxide synthase (iNOS) and Nox components such as p91phox and p22phox [19–22].

An increase in intracellular diacylglycerol (DAG) was suggested as a unifying hypothesis that could explain most forms of insulin resistance [8]. During hyperglycemia, de novo DAG synthesis occurs via the polyol pathway [23]. During fatty acid overload, DAGs are synthesized as intermediates in triacylglycerol synthesis [24]. DAG is an essential cofactor and activator of PKC isoforms such as PKC-*α*, PKC-*δ*, PKC-*ε*, PKC-*ζ*, and PKC-*θ* which catalyze serine phosphorylation of IRS-1 [25]. These PKC isoforms also interact with TLR and other components of the TLR4 pathway such as myD88 to promote NF-kB activation [26]. PKCs also activate Nox by promoting p47phox translocation to the membrane [27]. Nox isoforms such as Nox2, Nox3, and Nox4 have a critical role in hepatocyte, endothelial cell, skeletal muscle cell, and adipocyte insulin resistance [11, 12, 28–30], and Nox-derived ROS contribute to TLR4 activation and signaling [31].

iNOS plays a key role in skeletal muscle, adipose tissue, and hepatic insulin resistance [32–34]. Nox-derived superoxide anion (∙−O_2_) and iNOS-derived nitric oxide (NO) undergo a diffusion-controlled reaction to form the peroxynitrite anion (ONOO−) (Figure 1), and this is the only reaction that occurs at comparable or even higher rate than superoxide dismutase- (SOD-) catalyzed conversion of superoxide anion to hydrogen peroxide [35]. Peroxynitrite is a major contributor to insulin resistance. For example, treatment of cultured adipocytes with hypochlorous acid (HOCl) resulted in adipocyte peroxynitrite production, PKC-*θ*, IKK, JNK phosphorylation, IRS-1 serine 307 phosphorylation, and insulin resistance, and peroxynitrite inhibitors abolished the rest of these events [36]. The biological effects of peroxynitrite have largely been attributed to its direct oxidation of thiol groups, or its involvement in the formation of radicals that participate in reactions such as lipid oxidation and protein tyrosine nitration [33, 35]. However, the mechanism by which peroxynitrite induces PKC-*θ*, IKK, JNK, and IRS-1 serine 307 phosphorylation may at least partly involve singlet oxygen-mediated ER stress because (i) peroxynitrite is involved in singlet oxygen formation (Sections 7 and 8), (ii) singlet oxygen induces ER stress (Section 10), and (iii) ER stress activates PKC-*θ*, IKK, and JNK [34, 37, 38].

## 5. The Receptor for Advanced Glycation End Products (RAGE) Mediates Similar Effects as TLR4

Serum advanced glycation end products (AGEs) are an independent determinant of insulin resistance as determined by the homeostatic model assessment method (HOMA-IR) in both males and females [39]. AGEs signal via the RAGE receptor, which, like TLR4, induces the recruitment of myD88 and TIRAP, and downstream signaling via NF-kB upon phosphorylation of the cytoplasmic domain of RAGE by PKC-*ζ* [40]. RAGE signaling is associated with a positive autoregulatory loop that perpetuates NF-kB activation, since NF-kB increases the expression of RAGE [41] and the RAGE-ligand, high mobility box protein [42]. Thus, AGE-RAGE signaling is a key player in endothelial cell dysfunction and adipocyte insulin resistance [40, 41]. Apart from AGEs, a high concentration of uric acid also induces endothelial dysfunction through the RAGE receptor [42].

## 6. Elevated Singlet Oxygen Production Precedes Insulin Resistance and Diabetes

According to recent reports, plasma levels of two hydroxy-octadecadienes (HODES) specifically derived from singlet oxygen-mediated linoleic acid (LA) oxidation, namely, 10-hydroxy-8(E), 12(Z)-octadecadienoic acid and 12-hydroxy-9(Z),13(E)-octadecadienoic acid, rather than two HODES specifically derived from free radical LA oxidation, namely, 13-hydroxy-9(E),11(E)-octadecadienoic acid and 9-hydroxy-10(E),12(E)-octadecadienoic acid, are suitable biomarkers for predicting insulin resistance and type 2 diabetes in humans [2, 43]. Further, Tsumura Suzuki obese diabetes (TSOD) mice on a high-fat diet had significantly higher singlet oxygen-associated fatty acid oxidation products than control mice at week 5, ahead of significant differences in free radical oxidation-derived products and insulin resistance at week 8 [3]. Thus, it was suggested that excessive singlet-oxygen formation occurs as an early event in the pathogenesis of insulin resistance and type 2 diabetes and that singlet oxygen may be directly or indirectly involved in initiating these disorders [3].

## 7. Diverse Cell Types, Including Insulin-Responsive Cells, Produce Singlet Oxygen

Murotomi et al. [3] suggested that activated neutrophils were responsible for the elevated plasma levels of singlet oxygen-associated LPO products in TSOD mice, through the myeloperoxidase-HOCl-H_2_O_2_ system. However, as recently reviewed, singlet oxygen can be generated through many types of reactions involving molecules that are found in virtually all cell types [44], which is not consistent with this ROS being produced just by leukocytes. Interestingly, singlet oxygen is now recognized as an important signaling molecule in plant cells, as a result not only of its photodynamic formation in chloroplasts but also by dark reactions in nonphotosynthetic cells, even in roots, in response to wounding and other stresses [45]. Peroxisomes, mitochondria, and the nucleus are major intracellular regions of such plant cell singlet oxygen formation in the dark [45]. Although the mechanism of formation of singlet oxygen under such conditions is unknown, this may at least partly involve the reaction of peroxynitrite and hydrogen peroxide, because this reaction produces singlet oxygen [46] and peroxisomes are a site for both hydrogen peroxide and peroxynitrite formation in plant cells [47]. Singlet oxygen was also reported to be generated not only by cultured tumor cells but also by the cell-free culture medium upon the addition of hydrogen peroxide, by a process involving the formation of excited carbonyls [48]. Singlet oxygen formation has been demonstrated in enterocytes [49], endothelial cells [50], and hepatocytes [49, 51]. During liver ischemia-reperfusion injury, acute hepatocyte oxidative stress can occur independently of Kupffer cells, the resident macrophages [52], and hepatocyte oxidative stress produces cytokines and chemokines that activate the latter [53], which is a major source of singlet oxygen prior to neutrophil activation [54]. Such a sequence of hepatocyte-Kupffer cell-neutrophil oxidative stress and singlet oxygen formation might occur during nutrient overload and the development of hepatic insulin resistance. Cytochrome P450 2E1, whose protein levels are 10 times higher in hepatocytes than in Kupffer cells [55] and whose expression is induced by xenobiotics as well lipid overload [55, 56], is an important contributor to singlet oxygen formation by liver microsomes [57].

## 8. The Importance of Peroxynitrite Anion in Singlet Oxygen Formation during the Pathogenesis of Insulin Resistance

**Figure 2:**
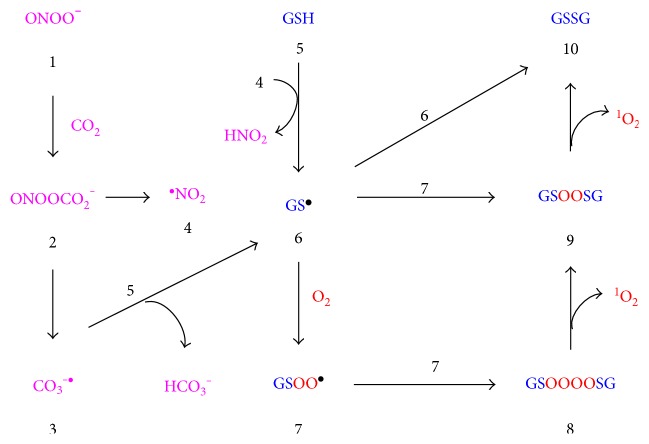
Formation of singlet oxygen (^1^O_2_) as a result of the facile conversion of peroxynitrite (1) to nitrosoperoxycarbonate (2), decomposition of the latter into carbonate and nitrogen dioxide radicals ((3) and (4), resp.) that convert glutathione (5) to glutathyl radical (6) [35, 60], reaction of (6) with oxygen to form peroxysulphenyl radical (7), and further reactions of the latter by a Russel-type mechanism to afford tetroxide (8), peroxide (9), oxidized glutathione (10), and ^1^O_2_ [44, 61]. Oxidized glutathione (10) can also be formed by the direct combination of two glutathyl radicals (6).

The reaction between peroxynitrite and hydrogen peroxide may produce more singlet oxygen than the neutrophil-associated reaction between hypochlorous acid and hydrogen peroxide in vivo because hypochlorous acid is very reactive with other biomolecules [58]. The related reaction between nitric oxide and hydrogen peroxide was also found to release large amounts of chemiluminescence due to singlet oxygen, and this reaction was suggested to be involved in nitric oxide-mediated cell killing [59]. Since insulin-responsive cells upregulate Nox and iNOS expression and the resultant formation of nitric oxide, peroxynitrite, and hydrogen peroxide under conditions relevant to insulin resistance (Sections 4 and 5), singlet oxygen should be generated under such circumstances. Peroxynitrite also reacts with various other molecules to generate singlet oxygen, as recently reviewed [44]. For example, as illustrated in Figure 2, the reaction of peroxynitrite (1) with CO_2_ forms nitrosoperoxycarbonate (2) which decomposes to reactive carbonate and nitrogen dioxide radicals ((3) and (4), resp.) that readily convert glutathione (5) to glutathyl radical (6) [35, 60]. Glutathyl radicals reacts with oxygen to generate peroxysulphenyl radical (7) which, via tetroxide species (8) and peroxide (9), generates ^1^O_2_ and glutathione disulfide (10) [61]. Since glutathione is one of the major peroxynitrite sinks [60], such reactions limit peroxynitrite-dependent free radical reactions while promoting singlet oxygen production.

## 9. Singlet Oxygen Generated near the Plasma Membrane May Induce Peroxynitrite Formation and Further Singlet Oxygen Formation in Insulin-Responsive Cells via the Death Receptor Fas

When tumor cells are exposed to a low dose of extracellular photodynamically generated singlet oxygen, the latter activates the death receptor Fas, which signals to upregulate Nox and NOS, resulting in peroxynitrite and H_2_O_2_ formation, and a “massive increase in secondary singlet oxygen” [62]. A similar phenomenon may contribute to endothelial cell dysfunction, because (i) endothelial cells express the Fas receptor [63]; (ii) endothelial cells may generate extracellular singlet oxygen because hydrogen peroxide and peroxynitrite formed in them can cross the plasma membrane [35]; (iii) treatment of endothelial cells with 3-morpholinosydnonimine (SIN-1), a peroxynitrite donor, increased iNOS expression via NF-kB and thus established a positive feedback loop for peroxynitrite formation [64]; (iv) high glucose-induced, Nox-mediated endothelial cell dysfunction is exacerbated by myeloperoxidase, which utilizes endothelial cell-derived hydrogen peroxide to generate hypochlorous acid [65], and should thus produce extracellular singlet oxygen by the reaction of the latter with hydrogen peroxide; and (v) apart from a direct effect of singlet oxygen on the endothelial cell Fas receptor, singlet oxygen may oxidize LDL to produce oxidized LDL, which signals via the Fas receptor to induce endothelial cell apoptosis accompanied by activation of MAP and Jun kinases [63]. In fact, the already mentioned (Section 4) hypochlorous acid-mediated, peroxynitrite-dependent induction of insulin resistance in cultured adipocytes [36] may involve a similar mechanism, whereby adipocyte-derived H_2_O_2_ reacts with HOCl to generate singlet oxygen, which then activates Fas. The latter, which has been shown to induce adipocyte insulin resistance [66], will then induce iNOS and Nox. The induction of iNOS activity in response to Fas activation in hepatocytes was shown to be a mechanism to reduce apoptosis and enhance survival [67]. Although the foregoing examples assume the activation of Fas by extracellular singlet oxygen, the same effects might generally result from singlet oxygen generated on either side of the plasma membrane, since singlet oxygen photodynamically generated near the plasma membrane was found to induce endothelial cell apoptosis [68]. In hepatocytes, Fas activation also promotes CYP2E1 activity [69], which is an important source of singlet oxygen [57].

## 10. Singlet Oxygen Formation in the Endoplasmic Reticulum Induces ER Stress

Endoplasmic reticulum stress is an important contributor to adipose tissue and hepatic insulin resistance, through multiple mechanisms including (i) activating JNK and p38, (ii) inducing the pseudokinase tribble 3 (TRB3), which prevents insulin-induced Akt phosphorylation, and (iii) upregulating protein tyrosine phosphatase B (PTPB), a negative regulator of the insulin receptor [34, 70, 71]. The ER stress-inhibiting chaperone tauroursodeoxycholic acid (TUDCA) has been shown to improve insulin signaling in both mice and humans [72, 73].

Singlet oxygen photodynamically generated within the ER induces calcium efflux and ER stress [74]. Cytochrome P450 2E1 (CYP2E1), which is mainly located in the ER, is a strong producer of superoxide [56] and also produces peroxynitrite even in the absence of iNOS [75]. Since the ER constantly produces H_2_O_2_ during protein folding [76], the reaction of CYP2E1-derived peroxynitrite with H_2_O_2_ to generate singlet oxygen and thereby induce ER stress is highly likely. CYP2E1 also generates singlet oxygen by a mechanism independent of peroxynitrite [57]. This protein also promotes lipid peroxidation, and its expression correlates with lipid peroxidation in obese patients [56, 77]. CYP2E1 expression is induced by JNK [78], which may be activated by pathways such as TLR4 or Fas [Sections 4 and 9], and CYP2E1 in turn strongly activates JNK [78]. Interestingly, adipocytes and hepatocytes strongly express CYP2E1 [78] and are prone to ER stress [34], while skeletal muscle cells only weakly express this protein [79, 80] and are less prone to ER stress [34]. There are both iNOS-dependent and iNOS-independent pathways for ER stress in the adipose tissue and liver, and silencing iNOS and abolishing the residual ER stress completely abolishes insulin resistance in these organs [34]. The iNOS-independent ER stress can be well explained by CYP2EI in hepatocytes and adipocytes. Whole body CYP2E1 knockout protected mice from HFD-induced obesity and insulin resistance, and it especially improved insulin sensitivity in hepatic and adipose tissues but not skeletal muscle tissue [81].

## 11. Mitochondrial Singlet Oxygen Formation Damages Mitochondrial DNA

Mitochondria are a key site for peroxynitrite formation due to NO easily diffusing into them and reacting with superoxide formed as a result of electron leakage from the electron transport chain [35]. Peroxynitrite further promotes such electron leakage, resulting in the elevation of mitochondrial H_2_O_2_ [35, 82], thus setting the right conditions for singlet oxygen formation by these two reactive species. Hence, mitochondria should be a major site for peroxynitrite-dependent singlet oxygen formation. This is consistent with the findings that, in skeletal muscle cells, mitochondrial ROS and the resultant DNA damage and apoptosis contribute to insulin resistance [13], attenuating mitochondrial hydrogen peroxide emission prevents skeletal muscle insulin resistance [17], deletion of iNOS or addition of a peroxynitrite inhibitor prevents such mitochondrial DNA damage and insulin resistance [14, 34], and skeletal muscles from rats injected with LPS produced singlet oxygen, which was associated with increased Nox and iNOS activity, hydrogen peroxide and peroxynitrite formation, and enhanced mitochondrial lipid oxidation [83].

ER calcium efflux is another major contributor to mitochondrial ROS, since it causes mitochondrial calcium influx, which greatly enhances mitochondrial superoxide production, for example, in cardiomyocytes [76]. In hepatocytes, uric acid-induced activation of Nox preceded ER stress, which further induced mitochondrial ROS [84].

**Figure 3:**
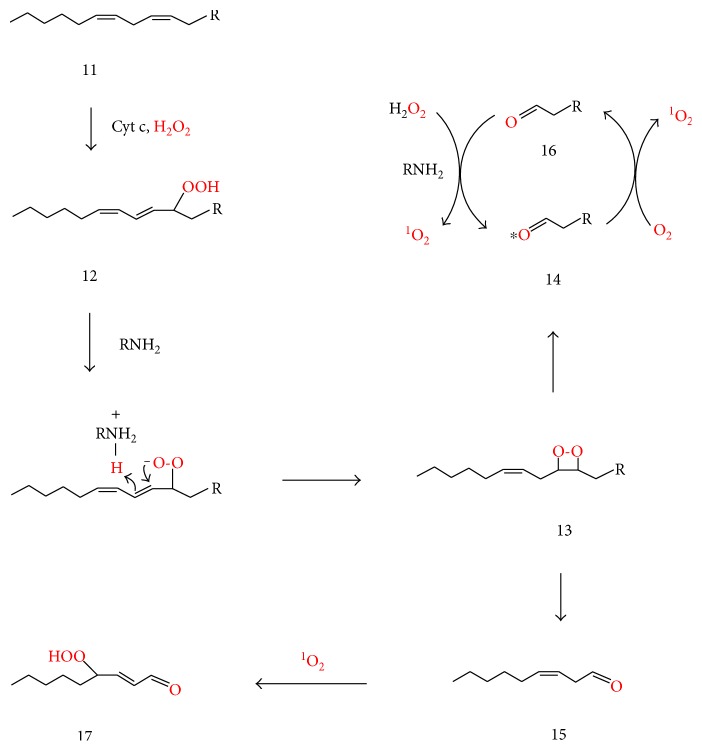
Proposed mechanisms for the mitochondrial formation of ^1^O_2_ and 4-hydroperoxy-2-nonenal (17) during the oxidation of a cardiolipin (11) (which bears an n-6 fatty acid). In the presence of H_2_O_2_, cytochrome c converts the latter to cardiolipin hydroperoxide (12) [85], followed by lysine-catalyzed rearrangement of the latter into dioxetane (13) [44] which decomposes into aldehyde (14) (oxononanoyl-cardiolipin in case the fatty acid moiety being oxidized is linoleic acid) and 3-Z-nonenal (15). The asterisk on carbonyl (14) indicates the excited (triplet) state, since dioxetane decomposition produces such carbonyls. Energy transfer from (14) to triplet oxygen converts the latter to ^1^O_2_ and the former to its ground state form (16), whose reaction with hydrogen peroxide regenerates (14) and forms singlet oxygen [44, 88, 90].

Increased mitochondrial H_2_O_2_ either as a result of peroxynitrite production or calcium influx may promote singlet oxygen formation by an additional pathway involving cardiolipin oxidation. In the presence of H_2_O_2_, cytochrome c acts as a cardiolipin-specific oxygenase, converting cardiolipin (11) to a cardiolipin hydroperoxide such as (12) (Figure 3) [85]. The decomposition of cardiolipin hydroperoxide generates triplet carbonyls that transfer energy to triplet oxygen and thus form singlet oxygen [86]. Formation of such triplet carbonyls from cardiolipin hydroperoxide (12) may partly involve the amine- (RNH_2_-) catalyzed conversion of the latter to dioxetane (13), whose decomposition affords an aldehydic cardiolipin (14) and 3(Z)-nonenal (15), either of which could be in the excited triplet state (Figure 3). The amine (RNH_2_) could be a lysine residue or the amino group of phosphatidylethanolamine or phosphatidylserine. Such amine-catalyzed conversion of the 13-hydroperoxide of linoleic acid (13-hydroperoxy-9Z, 11E-octadecadienoic acid, HPODE) to a dioxetane that yields hexanal was recently suggested [44] to explain the known reaction of lysine with HPODE to form N*ε*-(hexanoyl)lysine (HEL), which does not form by reaction of preformed hexanal with lysine in the absence of HPODE [87]. The Schiff base between hexanal and lysine was suggested to react with a second HPODE molecule to form HEL [44]. Formyl-lysine, a product analogous to HEL, is formed in a system containing formaldehyde, lysine, and H_2_O_2_ [88], where H_2_O_2_ (rather than a lipid hydroperoxide) reacts with the corresponding Schiff base. The fact that plasma HEL levels were significantly and positively correlated with fasting plasma glucose, serum insulin, and HOMA-IR in obese males [89] indicates that this kind of reaction is important in vivo.

Amines also catalyze a reaction between hydrogen peroxide and carbonyls (including sugars) to form singlet oxygen and excited carbonyls [44, 88, 90]. Such reactions might be responsible for the already-mentioned formation of singlet oxygen upon addition of hydrogen peroxide to a cell-free culture medium (Section 7) [48]. Accordingly, aldehyde (16), the nonexcited form of (14), may react with H_2_O_2_ to form singlet oxygen and to regenerate (14), thus amplifying singlet oxygen formation. In this way, any other aldehydes formed in the mitochondrion may participate in singlet oxygen formation. This aldehyde-amine-hydrogen peroxide-dependent mechanism of singlet oxygen formation may be equally important in the ER since it has been demonstrated in liver microsomes [88, 90], where CYP2E1 induces lipid oxidation in an environment favoring H_2_O_2_ formation [77].

## 12. Singlet Oxygen Oxidizes Low-Density Lipoprotein

Oxidized low-density lipoproteins (oxLDLs) were positively associated with HOMA-IR in young human adults independently of obesity in a longitudinal study [91]. Similar strong association between oxLDL levels and insulin resistance was obtained in a weight reduction study [92]. Endothelial cells mediate oxLDL formation by a peroxynitrite-dependent mechanism [93], indicating the potential involvement of singlet oxygen, since both singlet oxygen and oxLDL are strong precursors of insulin resistance [3, 92]. oxLDL can perpetuate the effects of HFD on endothelial cells, because its signaling via the lectin-like oxidized low-density lipoprotein receptor-1 (Lox-1 receptor) and TLR4 receptors initiates a positive autoregulatory loop for NF-kB activation and upregulation of Lox-1 receptor expression [94]. This also induces the expression of vascular cell adhesion molecule 1 (VCAM 1) and monocyte chemoattractant protein (MCP-1), which promote the recruitment of immune cells [94]. OxLDL signaling in adipocytes induces adipocyte insulin resistance through the activation of IKK, JNK, and NF-kB, even independently of further ROS formation [95], and this may involve the interaction of oxLDL receptor with CD36, resulting in CD36 association with the Src family tyrosine kinases Fyn and Lyn upstream of JNK [96]. oxLDL also reduces adiponectin secretion [97], and this affects systemic insulin resistance because adiponectin improves insulin sensitivity in endothelial cells, hepatocytes, and skeletal muscle cells [98].

As already discussed (Section 3), palmitate induces insulin resistance in insulin-responsive cells independently of neutrophils, but the latter are recruited to adipose tissue and promote diet-induced insulin resistance by producing myeloperoxidase and other proinflammatory substances. While palmitate lowers ROS formation by neutrophils [15], oxLDL induces neutrophil transmigration across microvascular endothelial cell monolayers and their subsequent degranulation especially after endothelial cell activation [99]. Extracellular hydrogen peroxide mediates the paracrine recruitment of neutrophils to wounded tissue [100]. Therefore, H_2_O_2_ produced by adipocytes, together with endothelial cell-derived oxLDL, may contribute to neutrophil infiltration and activation in adipose tissue.

## 13. Singlet Oxygen Increases Intracellular Ceramide Levels

Ceramide is a key contributor to insulin resistance by several mechanisms including, but not limited to, (i) activation of protein phosphatase 2A (PP2A) which dephosphorylates Akt, (ii) activation of the atypical PKC isoform *ζ* which inhibits Akt, and (iii) JNK activation [101].

Singlet oxygen induces the nonenzymatic conversion of sphingomyelin to ceramide in tumor cells, and there is an autocrine loop linking such ceramide increase to de novo ceramide biosynthesis [102]. There is a possibility that, at least the nonenzymatic mechanism, may be involved in ceramide formation in insulin-responsive cells such as skeletal muscle cells and adipocytes. Singlet oxygen may also contribute indirectly to ceramide accumulation via oxLDL-mediated decrease in adiponectin, since the latter has ceramidase activity [101], or by oxLDL-mediated sphingomyelinase activation [103]. Besides, ER stress and ceramide accumulation induce each other in a vicious cycle [101].

## 14. Singlet Oxygen Contributes to the Formation of Insulin Resistance-Promoting Reactive Carbonyl Species

The decomposition of lipid hydroperoxides affords highly reactive aldehydic products such as malondialdehyde (MDA), glyoxal, acrolein, 4-hydroperoxy-2-nonenal (HPNE), 4-hydroxy-2-nonenal (HNE), and 4-oxo-2-nonenal (ONE), which contribute to insulin resistance in various ways. Plasma MDA concentration positively correlates with insulin resistance [104]. ONE induces primary hepatocyte apoptosis through increased xanthine oxidase (XO) activity [105]. By promoting XO activity, ONE potentially contributes to the formation of uric acid, which induces endothelial dysfunction as well as hepatocyte ER stress [42, 84]. ONE reacts with lysine to form N*ε*-(4-oxononanoyl)lysine, an important ligand of Lox-1 receptor [106] and may thus make a major contribution to oxLDL-mediated insulin resistance. Protein-HNE adducts but not protein carbonyl levels were found to be related to intramyocellular lipid content and the severity of insulin resistance in humans [107].

Various mechanisms for the formation of some of the above-named aldehydes during free radical lipid oxidation have been proposed [108–110]. However, such purely free radical mechanisms do not adequately explain the generation of some lipid oxidation products in vivo. Notably, according to the free radical-dependent reactions, linoleic acid is not expected to be an important precursor of MDA [109], while, on the contrary, plasma linoleic acid was found to be an important precursor of this aldehyde [111]. Furthermore, the reaction of lysine with HPNE, a derivative of linoleic acid, was found to generate MDA by an unknown mechanism [112].

**Figure 4:**
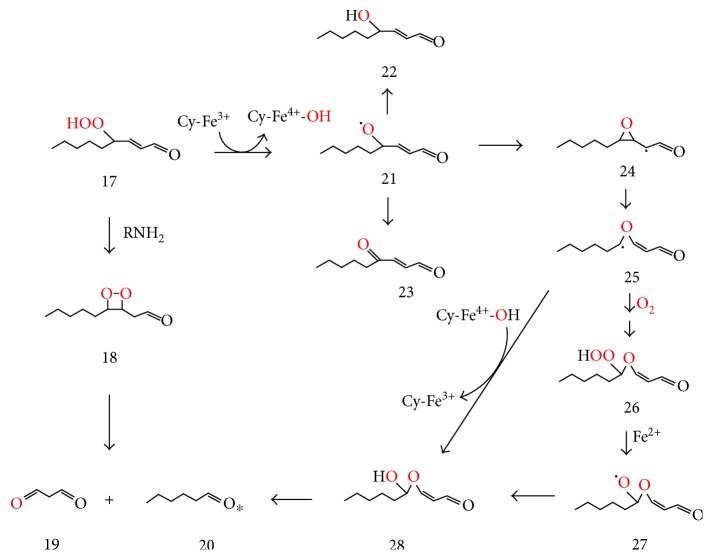
Suggested conversion of 4-hydroperoxy-2-nonenal (HPNE, (17)) to malondialdehyde (MDA, (19)), hexanal (20), 4-hydroxy-2-nonenal (HNE, (22)), and 4-oxo-2-nonenal (ONE (23)) through amine or metal ion-mediated processes.

HPNE (17) may be formed by the reaction of 3(Z)-nonenal (15) with singlet oxygen (Figure 3()). Similarly to the conversion of hydroperoxide (12) to dioxetane (13) (Figure 3()), HPNE (17) may be converted by an amine (RNH_2_) via dioxetane (18) to MDA (19) and hexanal (20) (Figure 4()), thus explaining the previously reported, lysine-mediated conversion of HPNE to MDA [112]. Inorganic ferrous ion (Fe^2+^) or organic ferric ions such as in cytochrome c (Cy-Fe^3+^) may alternatively convert HPNE (17) to the corresponding alkoxyl radical (21), which can abstract or lose a hydrogen to form HNE (22) or ONE (23), respectively, or cyclize to form epoxyalkyl radical (24), which rearranges to oxygen-stabilized vinyl ether radical (25) [113, 114]. The latter may react with oxygen and be converted via hydroperoxy-ether (26) and oxygen-centered radical (27) to hemiacetal (28). Vinyl ether radicals such as (25) also undergo direct conversion to hemiacetals such as (28) by oxygen rebound from the Cy-Fe^4+^ −OH (or Fe^3+^ −OH) pair, and the hemiacetals easily cleave to aldehydes such as MDA (19) and hexanal (20) [113–114].

Cholesterol oxidation generates two main aldehydic products, namely, cholesterol secosterol A and secosterol B [115]. Secosterol A potently inhibits endothelial nitric oxide synthase (eNOS) and neuronal nitric oxide synthase (nNOS), but not iNOS [116], and should therefore make an important contribution to endothelial dysfunction and insulin resistance, since eNOS promotes insulin sensitivity while iNOS promotes insulin resistance in humans [117].

**Figure 5:**
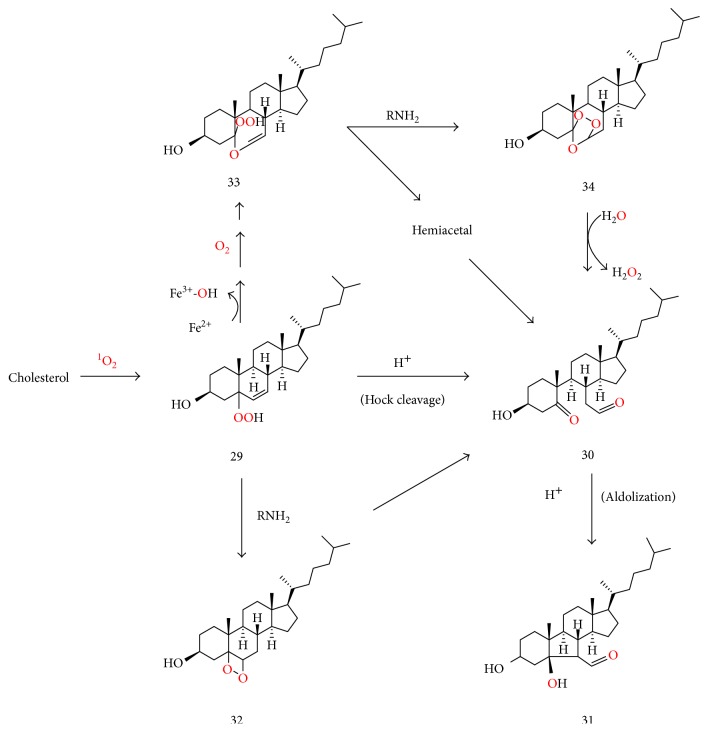
Suggested RNH_2_ and Fe^2+^ or cytochrome c-mediated conversion of cholesterol 5-hydroperoxide (29) to secosterol aldehyde A (30) via dioxetane (32), hydroperoxyvinyl ether (33) and the corresponding vinyl hemiacetal, or secondary ozonide (34). Although Hock cleavage of (29) also yields (30), the acidic conditions for Hock cleavage facilitate very rapid aldolization of (30) to form secosterol B (31).

Singlet oxygen but not free radical oxidation readily converts cholesterol to cholesterol-5-hydroperoxide (29), which, under acidic conditions, undergoes Hock cleavage to form cholesterol secosterol aldehyde A (30), followed by rapid acid-catalyzed aldolization of the latter to form secosterol B (31) (Figure 5), so that secosterol A is only detected as a minor product under such conditions [115, 118]. On the other hand, ozonolysis of cholesterol affords secosterol A as the main product [115, 118, 119]. Both secosterol aldehydes A and B have been isolated in significant quantities from various human tissues and LDL, confirming the importance of singlet oxygen, ozone, and/or an ozone-like oxidant in in vivo lipid oxidation [115, 118, 119]. These aldehydes were also detected in the plasma and other tissues of normal mice, further supporting the formation of singlet oxygen in endothelial cells and other cell types independently of leukocyte activation and inflammation [120]. The fact that secosterol A is a major product in vivo was interpreted as evidence for endogenous ozone formation [115, 118, 119]. Potential mechanisms for the antibody- or amino acid-catalyzed endogenous ozone formation in the presence of singlet oxygen have been proposed [119, 121]. However, Tomono et al. [122] reported that almost equal amounts of secosterols A and -B were formed in vitro from cholesterol oxidation by human myeloperoxidase (MPO) independently of antibody involvement and concluded that, in this case, singlet oxygen and possibly another oxidant, but not ozone, mediated the formation of both secosterols A and B.

As suggested in Figure 5, the amine-catalyzed decomposition of cholesterol 5-hydroperoxide (29) affords dioxetane (32) whose decomposition generates secosterol A (30) under nonacidic conditions. Moreover, cytochrome c-mediated conversion of cholesterol 5-hydroperoxide (29) to the corresponding alkoxyl radical and epoxyalkyl radicals was recently reported [123]. The said epoxyalkyl radical (not shown) may be converted to secosterol A (30) analogously to the conversion of epoxylakyl radical (24) to aldehydes (19) and (20) in Figure 4. It would not be unreasonable to postulate that hydroperoxyvinyl ether (33) (formed analogously to (26) in Figure 4) may undergo amine-catalyzed conversion to secondary ozonide (34), analogously to the amine-catalyzed formation of dioxetanes such as (32). Hydrolysis of secondary ozonide (34) then affords secosterol A (30) [124]. Thus, a combination of singlet oxygen, a metal ion, triplet oxygen, and an amine might act as an ozone-like oxidant.

## 15. Conclusion

Insulin resistance is a major precursor of diabetes and cardiovascular diseases, whose etiological pathways deserve attention. This review has highlighted the pathways of formation of singlet oxygen in insulin-responsive cells and how this ROS contributes to insulin resistance through ER stress, mitochondrial DNA damage, and the formation of oxLDL and bioactive aldehydes such as MDA, HNE, ONE, and cholesterol secosterol aldehydes A and B. Strategies for the prevention or management of insulin resistance may need to include dietary singlet oxygen quenching antioxidants but only as part of a multipronged approach that also targets events both upstream and downstream of singlet oxygen formation, such as inhibiting TLR4 signaling, detoxification of bioactive aldehydes, and inhibiting ER stress. Nevertheless, there is a need for more studies to directly determine the relative contribution of singlet oxygen to insulin resistance vis a vis other reactive oxygen and nitrogen species.
